# Implementing a Single-Session Nurse-Led Assessment Clinic into a Gender Service

**DOI:** 10.1089/trgh.2017.0050

**Published:** 2018-04-24

**Authors:** Donna M. Eade, Michelle M. Telfer, Michelle A. Tollit

**Affiliations:** ^1^ Department of Adolescent Medicine, Royal Children's Hospital Gender Service, Melbourne, Australia.; ^2^ Murdoch Children's Research Institute, Melbourne, Australia.; ^3^ Department of Pediatrics, The University of Melbourne, Melbourne, Australia.; ^4^ Melbourne Graduate School of Education, The University of Melbourne, Melbourne, Australia.

**Keywords:** access to care, adolescence, clinical care, gender dysphoria, health systems, transgender/trans sexual

## Abstract

The Royal Children's Hospital Gender Service offers support, assessment, and medical care to transgender and gender diverse children and adolescents in Victoria, Australia. Referrals have rapidly increased leading to extended wait times. In response, a single-session nurse-led assessment clinic (SSNac) was introduced as the clinical entry point to the service, during which a biopsychosocial assessment is undertaken, and information, education, and support are provided. Outcomes of the SSNac include a significant reduction in wait times and a timely clinical triage system. This article documents the creation and implementation of SSNac to offer a template for use in other gender services.

## Introduction

As awareness of gender diversity increases, so has the number of children and adolescents presenting to gender services in Australia and internationally.^1–3^ This growing demand has resulted in increased wait times for the assessment required to access medical transition pathways. Novel approaches to reduce wait time, enable timely clinical support, and provide education to patients and their families are required. This article describes a clinic developed within the Royal Children's Hospital Gender Service (RCHGS), Melbourne, Australia, which meets these objectives.

### Context

The RCHGS is currently the largest multidisciplinary service in Australia for transgender and gender diverse (TGD) children and adolescents. The service aims to improve the physical and mental health and well-being outcomes for this group. Any child or adolescent up to 17 years, who resides in Victoria, Australia, whose gender identity differs to their birth-assigned sex or who have concerns regarding their gender identity, can be referred to the RCHGS for assessment, support and a medical pathway should they require treatment.^4^

The RCHGS has seen TGD children and adolescents since 2003, with referrals to the service rising rapidly in the past 5 years from 18 in 2012 to 220 in 2016.^5^ In 2015, the RCHGS formalized the service and multidisciplinary team in response to the expanding wait list and increasing evidence that TGD young people have considerably higher rates of depression, anxiety, self-harm, and attempted suicide than their cisgender peers,^6–9^ which can, in part, be mitigated by accessing gender-affirming care.^10,11^ Included in the multidisciplinary team is a clinical nurse consultant (CNC), who typically is an advanced practice nurse with masters qualifications, and has advanced nursing knowledge, skills, and attributes relevant to their field with a broad scope of practice.^12,13^ At RCHGS, the CNC facilitates the development of advanced strategies and family-focused services to provide timely support and treatment for TGD children and adolescents.

## The Single-Session Nurse-Led Assessment Clinic

In 2016, referral numbers to the RCHGS escalated, resulting in a wait time of up to 14 months for an initial clinical consultation. In response to the lengthy wait time, the single-session nurse-led assessment clinic (SSNac), was introduced as the entry point to the RCHGS model of care. In the context of this article, wait time refers to the maximum amount of time from when a referral is accepted by the RCHGS to when a patient accesses care at their initial appointment.

The SSNac compromises of a 90-min, face-to-face, single-session consultation for TGD patients aged 8–17 years and their primary caregiver(s). The SSNac is led by the CNC who undertakes a biopsychosocial assessment^14,15^ of newly referred patients. As an outcome of this consultation, the CNC triages patients according to their clinical urgency and provides individualized support, education, and linkage to community-based services to help meet the needs of patients and their families.

A single-session clinical consultation offers a timely and effective approach that can be implemented to suit the service context.^16,17^ Although not a therapeutic model in a traditional sense, a single-session model is a useful way to educate and assist a patient and their caregivers regarding their immediate needs. For instance, by meeting the patient and assessing their needs in a single-session clinical consultation, relevant support and interventions can be identified and recommended.

During a SSNac consultation, the CNC meets with the young person and their primary caregiver(s) with a focus on providing information about the service and linkages to relevant community-based services. The CNC spends 30–40 min alone with the patient, and conducts a confidential biopsychosocial youth assessment to identify appropriate triage pathways, and provide health information and immediate interventions appropriate to individualized care. This is followed by a joint consultation with the patient and their primary caregiver(s), where information and applicable recommendations are made, including links to local community support services, mental health services, school supports, services relevant to TGD populations, peer support groups, and events. A letter summarizing recommendations is then sent to the patient and their referring general practitioner.

The SSNac's gender-affirming, youth-focused approach aims to be responsive to the needs of TGD young people.^9,18,19^ For example, all young people seen in SSNac are asked their preferred name and pronouns and whether they would like these to be used. From the outset of the consultation, the CNC explains what the service provides and how it may assist their health needs. Patient confidentiality is explicitly discussed. Developing patient rapport and enabling engagement to facilitate a positive, safe experience with the service for the young TGD patient are central to the success of the SSNac.

To effectively assess the needs of TGD adolescents, a youth-relevant framework is required.^18,19^ The HEADSS, acronym for “home, education, activities, drug and alcohol use, sexuality and suicide,” can be used for this purpose.^15^ The HEADSS is an adolescent biopsychosocial assessment framework for ascertaining risk and protective factors and is useful for identifying areas requiring health education and additional support appropriate to being a young TGD person. The HEADSS is used in each SSNac consultation.

The SSNac model provides opportunities for interventions to be tailored to meet the needs of individual adolescents. As an example, during the HEADSS assessment, many birth-assigned females who have commenced menses report dysphoria associated with menstruation and can be offered immediately available options for menstrual suppression such as oral norethisterone. Young people who wish to proceed with their multidisciplinary assessment for a medical pathway are provided education and links to support and this may help prepare them for the next phase of their assessment.

Through the SSNac, parents are also provided information and links to assist with their needs. The SSNac provides an opportunity for the caregiver(s) to ask questions and gain knowledge into community-based services that can assist them as well as their child, such as parent support groups and counseling services. The SSNac may also facilitate patient and parent readiness for entering the subsequent multidisciplinary assessment for a medical pathway and empower parents and young people to access resources and community-based supports earlier.

## Changes Since Introduction of SSNac

### Reduced wait time

Introduction of the SSNac and recruitment of a CNC has significantly reduced the wait time for clinical service access at RCHGS. Through SSNac, six additional appointments are provided each week. Patients now receive more timely access to care, as the wait time for a face-to-face consult before the introduction of the SSNac was 14 months, whereas they are now seen in SSNac within 4 months of their referral (Table 1). In addition, as an outcome of one's SSNac consultation, patients requiring urgent triage are fast tracked and seen in the multidisciplinary assessment for a medical pathway within 2 months.

**Table 1.** **Wait Time for Initial Consultation at RCHGS**

Year	2015 (Pre-SSNac)	2016 (Post-SSNac)	2017 (Post-SSNac)
Referral numbers (*n*)	180	220	141^a^
Wait time (months)	14	4	4

^a^ Seven months from January 1 to July 30, 2017.

SSNac, single-session nurse-led assessment clinic.

### Timely triage system

**FIG. 1.**
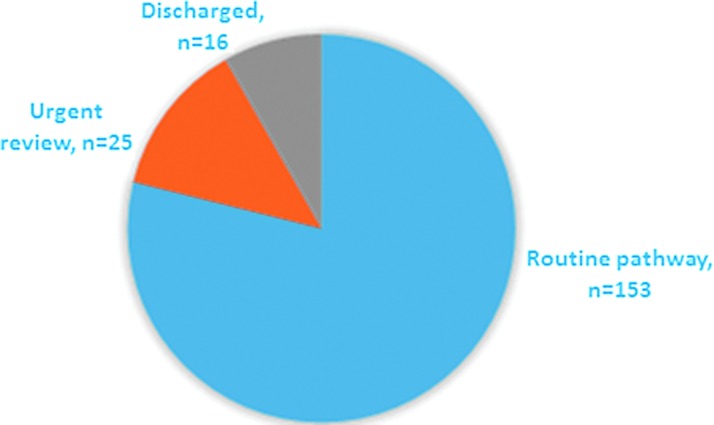
July 2016–2017 SSNac consultation triage outcomes. SSNac, single-session nurse-led assessment clinic.

The introduction of the SSNac enables the CNC to meet confidentially with the patients and clinically triage their care based on pubertal assessment and their hopes for medical transition. Consequently, at RCHGS, a fortnightly rapid review clinic has been established for patients seen in the SSNac who meet urgent triage criteria. This system enables those patients who will benefit most from puberty-blocking treatment to be fast tracked into the multidisciplinary assessment pathway to access treatment as required. Table 2 describes the triage criteria used at RCHGS, after participation in SSNac. Alternatively, patients not meeting urgent triage criteria are triaged from SSNac into the routine multidisciplinary assessment for a medical pathway and they are allocated their medical pathway appointments according to referral order. Through the SSNac, they are provided with general and individualized education and information on the medical pathways available, and relevant options for medical transition. Figure 1 summarizes the triage outcomes for SSNac for a 12-month period. Of the 194 patients who accessed care through SSNac, 8.2% were discharged, 12.9% were fast tracked for urgent multidisciplinary assessment for a medical pathway, and 78.9% patients entered the routine multidisciplinary assessment for a medical pathway.

**Table 2.** **Pubertal Status Markers Used for Fast Track Triage Criteria at RCHGS**

Birth-assigned males	Birth-assigned females
Age 10–12 years (consider 9–14 years)	Age 10–12 years (consider 8–13 years)
Tanner stage 1 or 2 based on testicular volume	Tanner stage 1 or 2 with no breast buds or minimal breast tissue present
Voice deepening absent or minimal	Premenarche

Patients who meet at least one of the mentioned criteria are considered for fast track triage from SSNac into MDAC.

To date, the implementation of the SSNac has been driven and informed by increasing clinical demand with outcomes predominantly assessed through wait time for service. A further evaluation of the potential impact of provision of information, education, support, and guidance through this model is anticipated for completion in 2018.

## Conclusion

The SSNac is an innovative nurse-led model that has significantly assisted the RCHGS in meeting the complex needs of TGD children and adolescents. The SSNac is unique worldwide in terms of its application of the single-session approach to a nurse-led model that is located within a gender service.

The 2016 implementation of the SSNac has resulted in the wait time for patients entering the RCHGS being reduced from 14 to 4 months. With a more efficient and effective triage system and improved opportunities to provide information, education, and support made possible through this model, it is anticipated that TGD children, adolescents, and their families will experience improved health and well-being outcomes in the crucial early stages of seeking care. The SSNac may be a useful addition to other gender services working to ameliorate the distress experienced by TGD children and adolescents on wait lists for multidisciplinary gender services.
